# Crusted Hyperkeratotic Scabies: A Case Report

**DOI:** 10.7759/cureus.34520

**Published:** 2023-02-01

**Authors:** Juan Marcos Meraz Soto, Ramiro Aleksander Alvarado Motte, Paola Ramírez Carrillo, Alan Axel Meraz Soto, Valeria Bayón Villaseñor, Orly Cheirif Wolosky

**Affiliations:** 1 Dermatology, Xochicalco University, Tijuana, MEX; 2 Dermatology, Autonomous University of Baja California, Tijuana, MEX

**Keywords:** scabies, diagnosis of scabies, immunosuppression, topical corticosteroids, ivermectin, sarcoptes scabiei, crusted scabies

## Abstract

Crusted scabies is a rare form of classic scabies characterized by severe symptoms, mainly observed in immunosuppressed patients. This disease has been associated with a variety of health problems, such as delayed diagnosis, infection risk, and high mortality, mainly from sepsis. We report the case of a patient with hyperkeratotic scabies in the context of immunosuppression associated with malnutrition and the use of topical corticosteroids. Ivermectin is critical for successfully treating crusted scabies. However, a higher cure rate has been reported with the combination of oral ivermectin and topical permethrin. In our study, we chose to use a plan suitable for grade two scabies, resulting in a subtotal regression of the lesions. Crusted scabies is a highly contagious parasitic cutaneous disease, and there are few reports in the national and international literature. It is necessary to suspect this presentation form in order to establish a timely diagnosis and detect and treat associated comorbidities.

## Introduction

Crusted scabies (CS), also called hyperkeratotic or Norwegian scabies, is a rare form of classic scabies caused by the infestation of hundreds to thousands of mites of Sarcoptes scabiei [1, 2]. The scabies mite was previously known as Acarus scabiei DeGeer in 1778; later, the term Sarcoptes was established and became S. scabiei [3].

The World Health Organization classifies classical scabies as a neglected disease. Although there is limited epidemiological information on CS, it is estimated that up to 200 million people worldwide are infected with the classic form of this illness [4, 5]. It is a worldwide problem affecting all races and socio-economic groups. It frequently affects children but can occur at any age. It is common in overcrowded living conditions, such as nursing homes, prisons, and among the homeless. In developed countries, it can be presented as a small epidemic in situations like war and natural disasters [6].

CS mainly affects immunosuppressed patients, such as those infected with HIV or those who suffer from AIDS, Down syndrome, prolonged use of corticosteroids, and malnutrition, as well as those patients with sensory alterations and physical limitations [1, 7].

It is most likely to be spread through close, direct contact. However, indirect transmission through contaminated materials such as clothing and/or bedspreads is possible [8]. This disease is due to the poor capacity of the host to limit the proliferation of the etiologic agent, which can decrease the lymphocytic response of the host against this pathogen, resulting in the hyperinfestation of mites in the outermost layer of the epidermis, the stratum corneum, whose cycle of life has an average duration of 14 days and can be found in any of its stages (egg, larva, nymph, adult) [9]. The high concentration of mites in the stratum corneum stimulates the unbalanced production of keratin, which can affect any area of the skin's surface [7]. Based on body surface areas, skin crusting depth, prior occurrences, admissions, amount of skin cracking, and pyoderma, classification systems for CS have been devised [2, 10].

The lesions are characterized by inflammatory papules, vesicles, indurated nodules and pustules, yellowish crusts, scaling, and flaking. Commonly affected areas are the dorsal and ventral aspects of hands and feet, respectively, the extensor surfaces, and the subungual area, and they also tend to be pruritic [11, 12].

We present the case of a patient with crusted scabies in the context of immunosuppression associated with malnutrition and the use of topical corticosteroids.

## Case presentation

**Figure 1:**
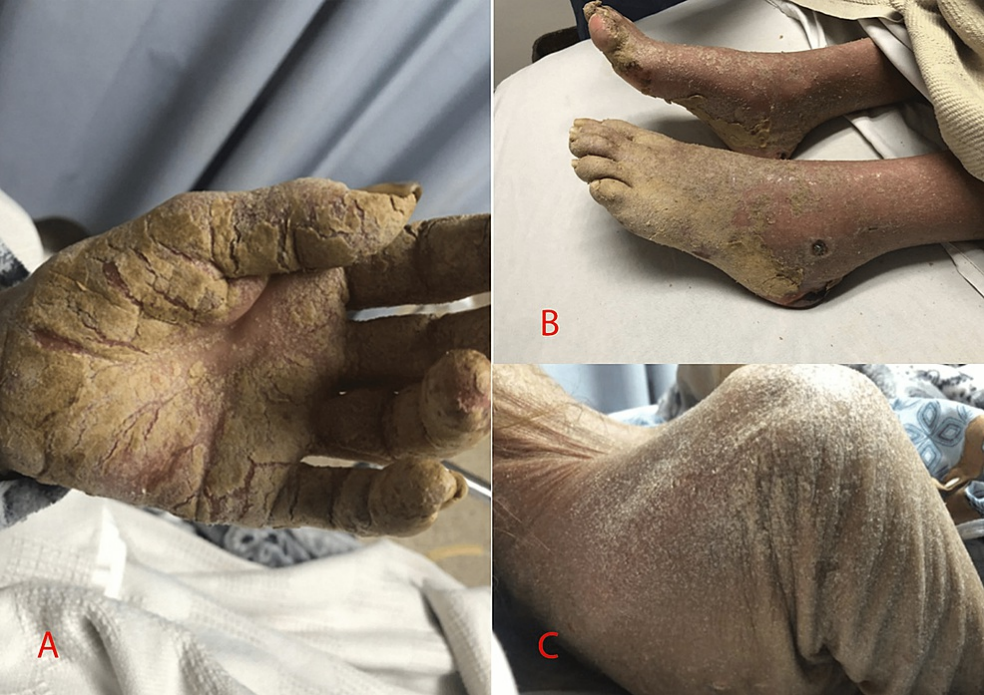
Physical findings: (1A) fissured hyperkeratotic lesions of the left hand; (1B) hyperkeratotic lesions on both feet; and (1C) flaky lesions on the posterior region of the thorax

We present the case of a 59-year-old female with a 30-year history of untreated type 2 diabetes. The patient arrived at the emergency room complaining of rib pain and a one-month evolution of generalized dermatosis, whereby she was hospitalized. An important precedent was the self-medicated use of topical corticosteroids for seven weeks to treat pruritus. On physical examination, the patient had a body mass index (BMI) of 16.4 kg/m2. Furthermore, multiple fissured hyperkeratotic lesions were observed, with a psoriasiform appearance, a yellowish-brown color, and a general distribution, although they mainly affected the hands (Figure 1A) and feet (Figure 1B). In addition, scaly lesions were observed distributed on both elbows, the anterior neck, and the posterior region of the thorax (Figure 1C). The keratotic plaques were firm and tender on palpation and were accompanied by pruritus.

**Figure 2:**
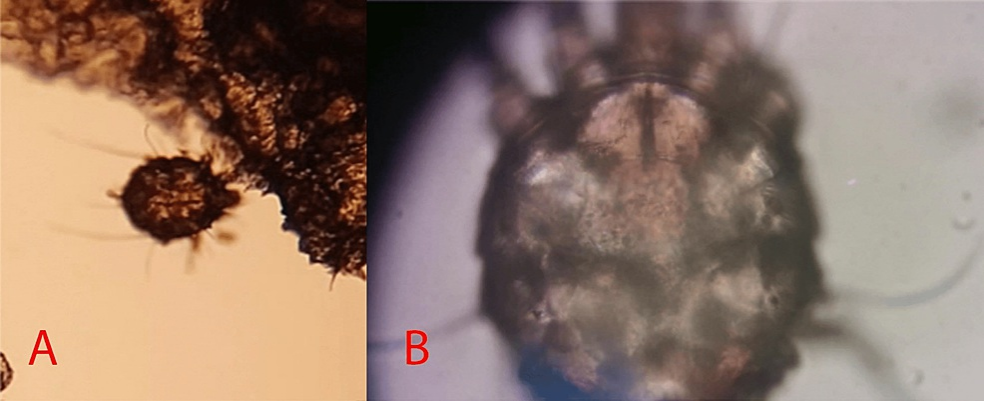
Microscopic examination: (2A) low-power microscopic visualization of the scabby plaque reveals the mite Sarcoptes scabiei; (2B) high-power microscopy examination of Sarcoptes scabiei

Direct scraping of the injured skin on the hand was performed with a number 10 scalpel blade, then the sample was placed on a slide and a drop of mineral oil was added (Muller's test). Microscopic analysis reveals the presence of an ovoid body (Figure 2A) similar to a turtle (idiosome), endowed with four pairs of short legs (Figure 2B), revealing infestation by Sarcoptes scabiei, which confirmed the diagnostic suspicion of CS.

**Figure 3:**
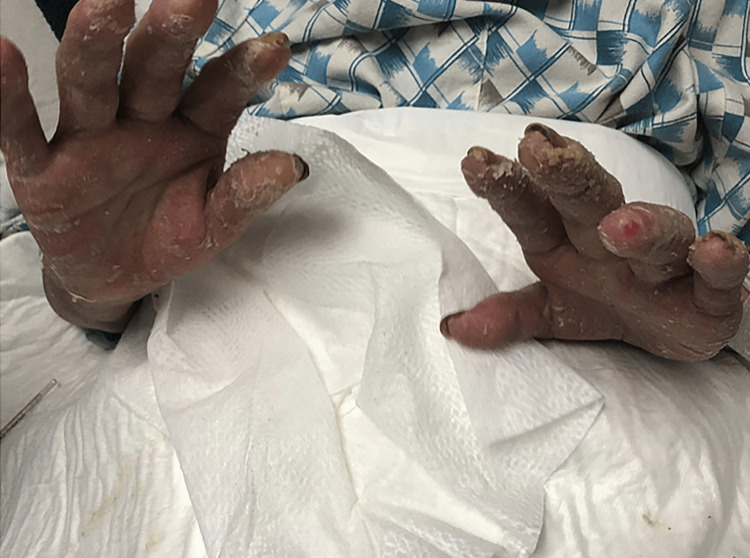
Subtotal regression of hand lesions after treatment

The patient was isolated, and an enzyme-linked immunosorbent assay (ELISA) was performed as part of the study protocol, ruling out HIV infection. Through laboratory tests, the presence of anemia and hyperglycemia became evident. The therapeutic intervention consisted of a pharmacological regimen with ivermectin at a dose of 200 micrograms per kilogram, given orally in five doses on days one, two, eight, nine, and 15 after diagnosis. Non-steroidal anti-inflammatory drugs and antihistamines were administered as part of symptomatic treatment. The clinicopathological evolution of the patient was remarkably favorable, resulting in a subtotal regression of the lesions (Figure 3), for which it was decided to continue with outpatient treatment.

## Discussion

We report the case of a patient with multiple risk factors (diabetes, malnutrition, and inappropriate use of topical corticosteroids) that favor host infestation by Sarcoptes scabiei. The diagnosis of crusted scabies could be made with a skin biopsy, dermoscopy, and direct microscopic visualization by a scraping technique also called Muller's test. The high sensitivity and specificity of direct microscopic visualization, the low level of complexity to carry out the test, and the low cost make it a better option for mite identification [2].

Evidence has demonstrated the association between inappropriate use of topical corticosteroids and localized decreased cell-mediated immune responses, promoting the spread and proliferation of the mite in this kind of scabies [13, 14]. On non-medical advice, our patient applied topical corticosteroids for seven weeks to treat pruritus, which exacerbated the mite infestation.

Ivermectin is critical to achieving a successful therapy for CS due to its mechanism of action, which causes paralysis of the mite through gamma-aminobutyric acid (GABA) receptor blockade [15]. However, a recent meta-analysis of 52 articles aimed to determine the efficacy and safety of antiparasitics reported a higher cure rate with the combination of oral ivermectin and permethrin, a topical scabicide [16]. The availability of this last-mentioned drug was a limitation in the treatment of our patient.

The therapeutic scheme with ivermectin at a dose of 200 mcg/kg will depend on the severity of the infestation. The classification system has assigned a grade according to its severity: first, second, and third grades [17]. We chose to use the plan suitable for grade two scabies, resulting in a subtotal regression of the lesions after concluding with the treatment.

Poor nutritional status, as measured by BMI, is not an independent risk factor for CS development; immunosuppression, poor hygiene, and exposure to the etiological agent are other significant variables [7]. As a result, in addition to pharmacological therapy, a strategy that targets these characteristics must be recommended to eliminate scabies infestations.

It is especially important to make an adequate diagnosis and provide timely treatment to avoid complications, such as bacterial superinfection, generally by Streptococcus pyogenes, secondary to scratching, which can trigger sepsis, streptococcal glomerulonephritis, and/or rheumatic fever [1, 18]. In addition, patients with CS are highly contagious transmitters, which contributes to outbreaks and the endemicity of the disease [18].

## Conclusions

CS is the least frequent presentation of scabies, and there are few reports in the national and international literature. There is relatively little worldwide epidemiological data available concerning this specific infection type. It’s necessary to suspect this presentation form of the disease to establish a timely diagnosis and detect and treat, as far as possible, associated comorbidities to avoid complications and reduce morbidity and mortality. This case study was carried out according to the principles proposed by the CARE (CAse REporting) guidelines.
